# Abnormal Size-Dependent Modulation of Motion Perception in Children with Autism Spectrum Disorder (ASD)

**DOI:** 10.3389/fnins.2017.00164

**Published:** 2017-03-29

**Authors:** Olga V. Sysoeva, Ilia A. Galuta, Maria S. Davletshina, Elena V. Orekhova, Tatiana A. Stroganova

**Affiliations:** ^1^Center for Neurocognitive Research (MEG Center), Moscow State University of Psychology and EducationMoscow, Russia; ^2^Autism Research Laboratory, Moscow State University of Psychology and EducationMoscow, Russia; ^3^Gillberg Neuropsychiatry Centre, University of GothenburgGothenburg, Sweden

**Keywords:** motion perception, spatial suppression, spatial facilitation, excitation/inhibition balance, autism spectrum disorders (ASD), children

## Abstract

Excitation/Inhibition (E/I) imbalance in neural networks is now considered among the core neural underpinnings of autism psychopathology. In motion perception at least two phenomena critically depend on E/I balance in visual cortex: spatial suppression (SS), and spatial facilitation (SF) corresponding to impoverished or improved motion perception with increasing stimuli size, respectively. While SS is dominant at high contrast, SF is evident for low contrast stimuli, due to the prevalence of inhibitory contextual modulations in the former, and excitatory ones in the latter case. Only one previous study (Foss-Feig et al., 2013) investigated SS and SF in Autism Spectrum Disorder (ASD). Our study aimed to replicate previous findings, and to explore the putative contribution of deficient inhibitory influences into an enhanced SF index in ASD—a cornerstone for interpretation proposed by Foss-Feig et al. (2013). The SS and SF were examined in 40 boys with ASD, broad spectrum of intellectual abilities (63 < IQ < 127) and 44 typically developing (TD) boys, aged 6–15 years. The stimuli of small (1°) and large (12°) radius were presented under high (100%) and low (1%) contrast conditions. Social Responsiveness Scale and Sensory Profile Questionnaire were used to assess the autism severity and sensory processing abnormalities. We found that the SS index was atypically reduced, while SF index abnormally enhanced in children with ASD. The presence of abnormally enhanced SF in children with ASD was the only consistent finding between our study and that of Foss-Feig et al. While the SS and SF indexes were strongly interrelated in TD participants, this correlation was absent in their peers with ASD. In addition, the SF index but not the SS index correlated with the severity of autism and the poor registration abilities. The pattern of results is partially consistent with the idea of hypofunctional inhibitory transmission in visual areas in ASD. Nonetheless, the absence of correlation between SF and SS indexes paired with a strong direct link between abnormally enhanced SF and autism symptoms in our ASD sample emphasizes the role of the enhanced excitatory influences by themselves in the observed abnormalities in low-level visual phenomena found in ASD.

## Introduction

ASD is a neurodevelopmental disorder diagnosed by behavioral impairments in three functional domains: social relatedness, social communication and stereotyped behavior with unusually narrow interests (American Psychiatric Association, 2013). Previous psychophysical and neurophysiological research indicates that the core symptoms of ASD are not limited to impaired social communication, but extend to atypical perceptual skills in different sensory modalities including vision (Davis et al., 2006; Simmons et al., 2009; Marco et al., 2011; Davis and Plaisted-Grant, 2015). Atypical low-level visual functions in individuals with ASD are especially interesting due to their strong reliance on primary pathophysiological mechanisms and lesser dependence on secondary factors, such as restricted social and perceptual experience and intellectual developmental disability that may affect complex visual perceptual skills in children with developmental disorders. Although basic visual functions, such as visual acuity and contrast sensitivity were proved to be generally preserved in ASD (Kéïta et al., 2010; Bölte et al., 2012), several low-level skills were found to be either super-optimal [e.g., Vernier acuity (Latham et al., 2013); temporal resolution (Falter et al., 2013); contrast sensitivity (Bertone et al., 2005)] or sub-optimal (e.g., binocular interactions Robertson et al., 2013; orientation sensitivity along cardinal axis, Sysoeva et al., 2016). Growing evidence for abnormalities in visual sensory processing in individuals with ASD has raised a question about underlying cellular and network mechanisms that cause alternations in the development course of the visual functions in this disorder.

Excitation/inhibition (E/I) imbalance in neural networks is now considered among the core neural underpinnings of autism psychopathology (Rubenstein and Merzenich, 2003; Lee et al., 2016). Data from animal models of ASD suggest a causal link between inhibitory circuitry malfunction at different level of brain hierarchy and various impairments in social cognition, perception and motor behavior (for review see Nelson and Valakh, 2015). The supportive evidence on deficient inhibition in the brain of individuals with ASD is starting to emerge from several lines of research. The neurophysiological index of E/I balance in neuronal population—high frequency (gamma-band) brain oscillations (Yizhar et al., 2011)—was found to be atypical in ASD individuals both in spontaneous brain activity (Orekhova et al., 2007) and during visual processing (Stroganova et al., 2012, 2015; Sun et al., 2012). A proton magnetic resonance spectroscopy studies reported significantly reduced concentration of main inhibitory neurotransmitter, γ-Aminobutyric acid (GABA) in several areas of autistic brain, i.e., basal ganglia, prefrontal, auditory, and motor cortex (Harada et al., 2010; Gaetz et al., 2014; Rojas et al., 2014). In neuro-typical people GABA concentration in visual cortex correlated with the psychophysical measure of the dynamics of binocular rivalry - duration during which one of the two competing images presented to each eye was fully suppressed from visual awareness (Robertson et al., 2016). Most importantly, this relationship was completely absent in ASD individuals. The authors hypothesized that de-coupling between binocular rivalry and GABA concentration in ASD may arise from perturbations in key components of the GABAergic pathway in primary visual cortex. Therefore, the E/I imbalance in ASD may be present not only in social, emotional, and language systems but also in purely sensory areas of the brain and might play an important role in different aspects of visual perception, for example, through disturbed divisive normalization of neural population activity (Rosenberg et al., 2015). Divisive normalization is the process by which neural responses are scaled according to the total amount of neural activity in the respective neural network and acts to optimize visual processing under changing stimulation conditions (Isaacson and Scanziani, 2011).

In particular, the balance between excitation and inhibition in visual cortex and the resulting differences in divisive normalization affect perception of visual motion. Studies of human perception of drifting gratings revealed at least two different motion-sensing mechanisms that operate over different spatial scales and visual contrast (Tadin and Lappin, 2005). Specifically, the phenomenon of spatial suppression (SS) refers to impoverished perception of motion direction while a participant is briefly presented with high-contrast stimuli of a large size (e.g., gratings covering more than 1–2 degree of visual angle). The SS predominantly occurs at high contrast (Pack, 2004; Paffen et al., 2005) and is thought to reflect center-surround antagonism in neurons at different levels of visual processing hierarchy including the primary visual cortex (V1) and the intermediate motion sensitive areas (V3 and MT). The SS allows visual system to segregate and spatially localize features of large high-contrast moving images (Sceniak et al., 1999). Conversely, low-contrast stimuli of relatively larger sizes favor the discrimination of motion direction—a phenomenon known as spatial facilitation (SF). The SF improves sensitivity for the near-threshold stimuli (Born and Bradley, 2005). The sign (i.e., facilitation or suppression) of the modulatory effect of size on motion discrimination thus depends on stimulus contrast with facilitation dominating for low-contrast stimuli and suppression for high-contrast ones (Tadin and Lappin, 2005).

Studies in psychiatric patients link altered SS and SF in the clinical populations to a weakened GABA-ergic inhibitory function in cortical networks (Betts et al., 2005, 2009, 2012; Tadin et al., 2006; Golomb et al., 2009). The SS was found to be reduced in patients recovered from major depressive disorder (MDD) and inversely correlated with the illness load measured as an amount of time a patient had been spent depressed (Golomb et al., 2009). Similarly, in patients with schizophrenia weakening of SS correlated with the negative symptom severity (Tadin et al., 2006). In line with numerous data on the age-related decline in cortical inhibition, Betts et al. (2005, 2009, 2012) reported a decreased SS indexes in old comparing to young participants. Notably, the results on SF at low contrast were less consistent across different clinical populations. For example, the SF index did not differ from normal values in patients with schizophrenia (Tadin et al., 2006) or in elderly subjects (Betts et al., 2005) but it was found atypically high in MDD patients, however, not related to illness load (Golomb et al., 2009).

Despite rather complex relationships between abnormalities in spatial suppression and spatial facilitation phenomena suggestive of difference in underlying neural causes, the psychophysical results in patients and elderly subjects has been interpreted as consequences of the same neural deficiency—reduced efficacy of GABA-ergic inhibition in visual system (Betts et al., 2005; Tadin et al., 2006; Golomb et al., 2009).

Meanwhile, animal studies (Sceniak et al., 1999; Angelucci and Bressloff, 2006) revealed that at least two neural mechanisms—inhibitory and excitatory influence on the classical receptive field from the surrounding neural population—operate simultaneously at any level of stimulus contrast. The specific sign of the modulatory effect driven by increasing stimulus size—spatial suppression or spatial facilitation—depends on the balance between excitation and inhibition in center-surround interaction in visual areas V1 and MT. Balancing result is qualitatively different for high- and low-contrast stimuli, with inhibition from the surround prevailing in the former and excitation in the latter case (Kapadia et al., 1999; Sceniak et al., 1999). Hence, theoretically we could expect that, under a pathological condition, the changes in the SS and the SF may occur either in parallel or separately, depending on the underlying neural cause. If driven exclusively by a compromised inhibition, which shifts the balance toward heighten excitation, the atypically reduced SS under high contrast should be accompanied by an abnormally strong SF under low contrast condition.

Whilst being well suited for targeted and specific testing of the putative deficit in divisive normalization in early visual areas in ASD (Rosenberg et al., 2015), the psychophysical phenomena of SS and SF in individuals with ASD were examined in only one previous study (Foss-Feig et al., 2013). Having considered the existing evidence on reduced efficacy of the inhibitory GABAergic system in ASD, Foss-Feig et al. hypothesized that children and adolescents with ASD would exhibit a decreased SS. Unexpectedly, the study's results were qualitatively different from the previous observations in people with schizophrenia and depression. Firstly, Foss-Feig et al. found that, compared to typically developing (TD) children, children with ASD had generally higher sensitivity to moving gratings—i.e., they needed relatively less time to distinguish direction of motion in high-contrast shortly presented displays of moving gratings of different sizes. This finding was surprising given a rapid visual-motion integration deficit frequently observed in children with ASD in series of studies applying a variety of behavioral paradigms (reviewed by Gepner and Féron, 2009). Another unexpected finding of the study was the presence of the undisturbed SS in children with ASD. On the other hand, the SF effect was abnormally strong in this group, especially at the largest size (6 degree of visual angle) of the low-contrast stimulus. Foss-Feig et al. (2013) attributed the heightened SF to weakening of the center-surround inhibition in autistic visual cortex, specifically in the area MT. The question remained unanswered is why putatively inefficient inhibition affected center-surround interactions at a low contrast, but not influenced them when overall contrast was high, i.e., under the experimental condition, which was optimal for revealing a putative weakening of surround suppression mechanisms.

The present study is, in part, a replication and an extension of the Foss-Feig et al. (2013) investigation, attempting to shed some light on the problems raised by their research. Firstly, as in the case with the majority of psychophysical studies of individuals with ASD, reported abnormalities might be elusive, poorly reproducible between different samples (Kaiser and Shiffrar, 2009), and therefore need independent confirmations. Secondly, the extent and direction of the effects that Foss-Feig et al. observed in individuals with ASD may depend on age and/or vary with severity of intellectual disability. Individuals with ASD recruited for Foss-Feig et al. study were highly intelligent, almost exceeding by their IQ scores typical individuals from their sample and thus representing a small minority of population with ASD generally characterized by intellectual difficulties (Charman et al., 2011). With this in mind, we included into our study ASD children with rather broad range of IQ. Thirdly, we attempted to separate the respective contribution of inhibitory and excitatory influences into SF psychophysical index—a cornerstone for interpretation proposed in the previous study. This has been done by measuring a correlation between the SS at a high contrast and the SF at a low contrast in the same participant. If, as it was assumed by Foss-Feig et al. (2013), inhibitory influences from the surround operate for very large stimuli even at a low contrast, we can expect that SF at a low contrast and SS indexes at a high contrast should be strongly interrelated. We anticipated that this would allow us to distinguish increased levels of excitatory influences from weakened inhibition from the surround in children with ASD.

## Methods

### Participants

Forty nine boys with ASD and 44 TD boys aged from 6 to 15 years were recruited at rehabilitation centers affiliated with the Moscow University of Psychology and Education and from the local community, respectively. The exclusion criteria were the presence of a known chromosomal syndrome (e.g., Down Syndrome, Fragile X syndrome), or other diagnosed neuropsychiatric disorder (e.g., epilepsy). All ASD participants were examined by the experienced psychiatrist, who confirmed the diagnosis of ASD based on the Diagnostic and Statistical Manual of Mental Disorder-5 criteria and an interview with the parents/caregivers. Additionally, parents of the majority of the children (33 ASD and 39 TD) filled in the Russian translation of the Social Responsiveness Scale for children (Constantino and Gruber, 2012). One child in the ASD group had a SRS raw score of 56, which is below the recommended cut-off for the presence of social difficulties (T scores of 59), but is in line with the reported sensitivity of the SRS (93%, Constantino and Gruber, 2012). All other ASD participants had SRS T-scores higher than 64. Two of the TD participants (5%) were above the 59 T-score cut-off, which is also in line with the previously reported specificity of the SRS (90%, Constantino and Gruber, 2012). All participants with ASD were verbal, but most of them (71%) had a history of speech delay (not a single word produced by age 2 and/or no phrases by age 3) according to the parental report and medical records. Participants's IQ was assessed during a separate visit using Kaufman Assessment Battery for Children K-ABC II (Kaufman and Kaufman, 2004) in all but one ASD child, who was unable to attend the assessment due to logistic reasons. In the majority of the participants (35 ASD and 40 TD) clinically relevant sensory behaviors were assessed using Sensory Profile Questionnaire (SPQ, Dunn, 1999). The SPQ questionnaire contains 125 items arranged into eight categories: auditory, visual, taste/smell, movement, body position, touch, activity level, and emotional/social. Based on these items, we calculated four quadrant scores (registration, sensory seeking, sensitivity and avoidance, Dunn, 1999). All children had normal or corrected to normal vision according to the available medical records.

Previous research suggests significant sex differences in etiological factors and the behavioral manifestation of ASD (Lai et al., 2015). To limit the influence of additional factors and to make the study sample more homogeneous, in the current study we investigated only male participants, leaving the question about generalizability of our results across genders for the future studies.

The investigation was approved by the local ethics committee of the Moscow University of Psychology and Education and was conducted following the ethical principles regarding human experimentation (Helsinki Declaration). All children provided their verbal consent to participate in the study and were informed about their right to withdraw from the study at any time during the testing. Written informed consent was also obtained from a parent/guardian of each child.

### Stimuli and paradigm

**Figure 1:**
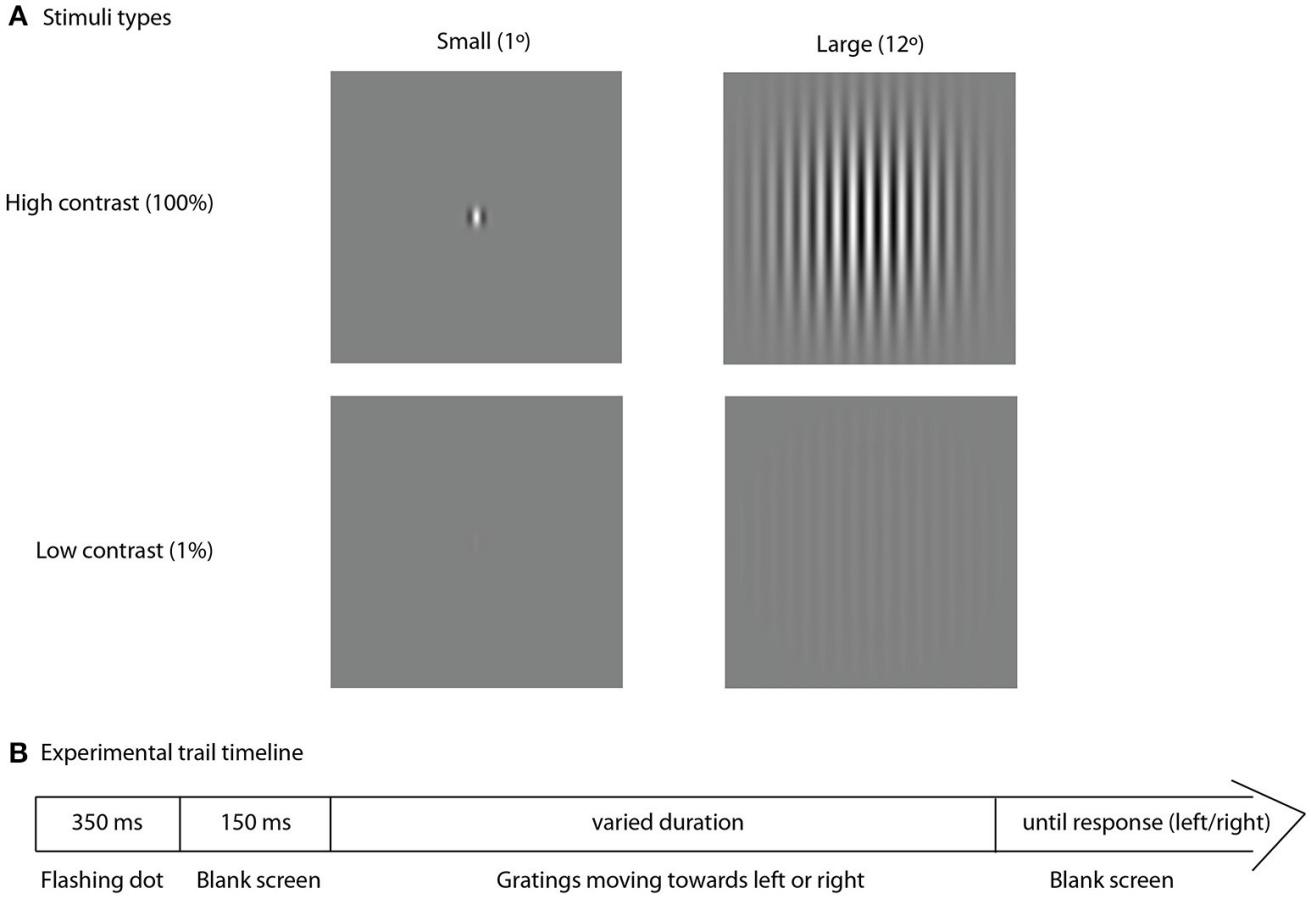
**Experimental trial**. Type of stimuli, used in the experiment **(A)** and schematic representation of the experimental trial timeline **(B)**.

Visual stimuli were presented using PsychoToolbox (Brainard, 1997), a free Matlab application. To assess spatial suppression we used an experimental approach similar to that described by Foss-Feig et al. (2013). Figure 1 represents the stimuli and experimental procedure. The stimuli consisted of the drifting vertical sine wave gratings (1 cycle/degree, 4 degrees/sec), covered by two-dimensional Gaussian envelope with the radius defining the stimulus size (small–1° or large–12°). Stimuli were presented at either low (1%) or high (100%) contrast in two separate blocks. Direction of motion (left or right) was determined randomly for each trial. Participants sat at 60 cm distance from the monitor (Benq XL2420T, 24″W LED, 1,920 × 1,080 resolution, 120 Hz). A research assistant, seated next to each participant, controlled for correct distance from the monitor, vertical head position and adequate task performance. Participants were asked to make an un-speeded two-alternative forced-choice response indicating the perceived right or left direction of motion by pressing the left or the right arrows on the keyboard, respectively. Inter-trial interval was 500 ms. In the beginning of each trial a central dot flickered at the screen (50 ms on, 50 ms off, 250 ms on, 150 ms off) followed by the stimulus presentation. The initial stimulus duration was set to 150 ms. The duration was further adjusted depending on participant's response using two (one for small and one for large stimuli) interleaved one-up two down staircases (8.3 ms step) that converged on 71% correct performance. The block continued until both staircases completed 9 reversals, typically lasting around 4 min. The thresholds were computed by averaging over the reversals, excluding the first two of each staircase. Most participants repeated the block two times (*N* = 37 in the ASD group and *N* = 40 in the TD group) and the thresholds obtained in the two experimental sessions were averaged for final analysis.

Before the main experimental sessions the experimental procedure was explained to each participant. Then they performed a short training session that included only 10 trials, but otherwise was identical to the main session. The experimenter carefully watched the performance and provided help when needed. If there were any concerns that participant did not understand the task, the explanation was repeated and the training session was re-administered.

### Analysis

To normalize the data distribution we calculated the common logarithm of the motion direction discrimination thresholds measured in milliseconds. In the low-contrast condition, distribution of the log-transformed motion direction discrimination thresholds did not deviate from normal in either group (Shapiro-Wilk test, all *p*'s > 0.16). In the high-contrast condition, the distribution of thresholds deviated from normal in the ASD group (*p* = 0.02 and *p* = 0.004, for small and large stimuli, respectively) yet not in the TD group (all *p*'s > 0.4). However, Skewness and Kurtosis were smaller than 1 for all stimuli, conditions and groups, suggesting applicability of parametric statistics. An Analysis of Variance (ANOVA) with the factors Size (Large vs. Small, within-subject factor) and Group (ASD vs. TD, independent factor) was used to examine main effects and their interaction. Planned *post-hoc* tests were used to follow up the significant ANOVA effects. The effect sizes were estimated using η^2^.

In the majority of the previous publications, the SF and the SS indexes were calculated as the difference between log-transformed motion direction discrimination thresholds for large and small stimuli under low- and high-contrast condition, respectively (Tadin and Blake, 2005; Tadin and Lappin, 2005; Tadin et al., 2006, 2011; Golomb et al., 2009; Foss-Feig et al., 2013). In this study we used an alternative way to extract the unique effect of increased stimulus size on motion discrimination. Specifically we computed the residual values of the thresholds for large stimulus size after partialling out the effect of threshold for the small stimuli using regression analysis. The SS and the SF indexes distributions did not deviate from normality in both groups (Shapiro-Wilk test, all *p*'s > 0.4) that justified the implementation of the parametric statistics in the current study (e.g., Pearson correlation coefficient). Pearson product-moment coefficients were used to investigate the correlations of motion direction discrimination parameters (discrimination thresholds, SF, and SS indexes) with age, IQ, and questionnaire measures (SPQ and SRS). Fisher *Z*-test was used to estimate group differences in correlation coefficients.

## Results

### Final sample

Nine ASD participants were unable to complete the psychophysical task, therefore, our final sample comprised 40 ASD and 44 TD boys. The information on participants included in the study is summarized in Table 1.

**Table 1:** **Participants demographics**.

	**TD Mean (*SD*), range**	**ASD Mean (*SD*), range**	**Statistics *t*, *p* values**
Age, years	10.8 (2.1), 6.6–15.1	10.1 (2.2), 6.3–15.1	*t* = 1.65, *p* = 0.103
IQ, KABC total score	119 (10), 94–141	90 (18), 63–127	*t* = 9.1, *p* < 0.0001
SRS Raw score	47 (24), 8–91	107 (21), 56–141	*t* = 11.3, *p* < 0.0001
SPQ1: Low registration	64 (6), 44–74	51(10), 25–70	*t* = 6.87, *p* < 0.0001
SPQ2: Sensation Seeking	108 (10), 81–125	94(17), 63–123	*t* = 4.54, *p* < 0.0001
SPQ3: Sensory sensitivity	86 (8), 63–98	74(10), 50–95	*t* = 5.97, *p* < 0.0001
SPQ4: Sensory avoidance	117 (13), 78–136	98(15), 72–128	*t* = 5.77, *p* < 0.0001

### Effect of age and IQ

Considering the wide age range of our participants (6–15 years of age), we checked for a possible age-dependence of the motion direction discrimination thresholds and the SS and the SF indexes in the TD and the ASD groups. Neither of these measures correlated significantly with the age in either group (all /*r*'s/ < 0.28, *p*'s > 0.08), suggesting relative stability of the observed effects during school years. There were no significant correlations between psychophysical measures (discrimination thresholds, SS, SF) and IQ (all /*r*'s/ < 0.20, all *p*'s > 0.24), pointing to an independence of the observed effects from the intellectual abilities in either the clinical or the control samples.

### Between-group differences

#### High-contrast stimuli

**Figure 2:**
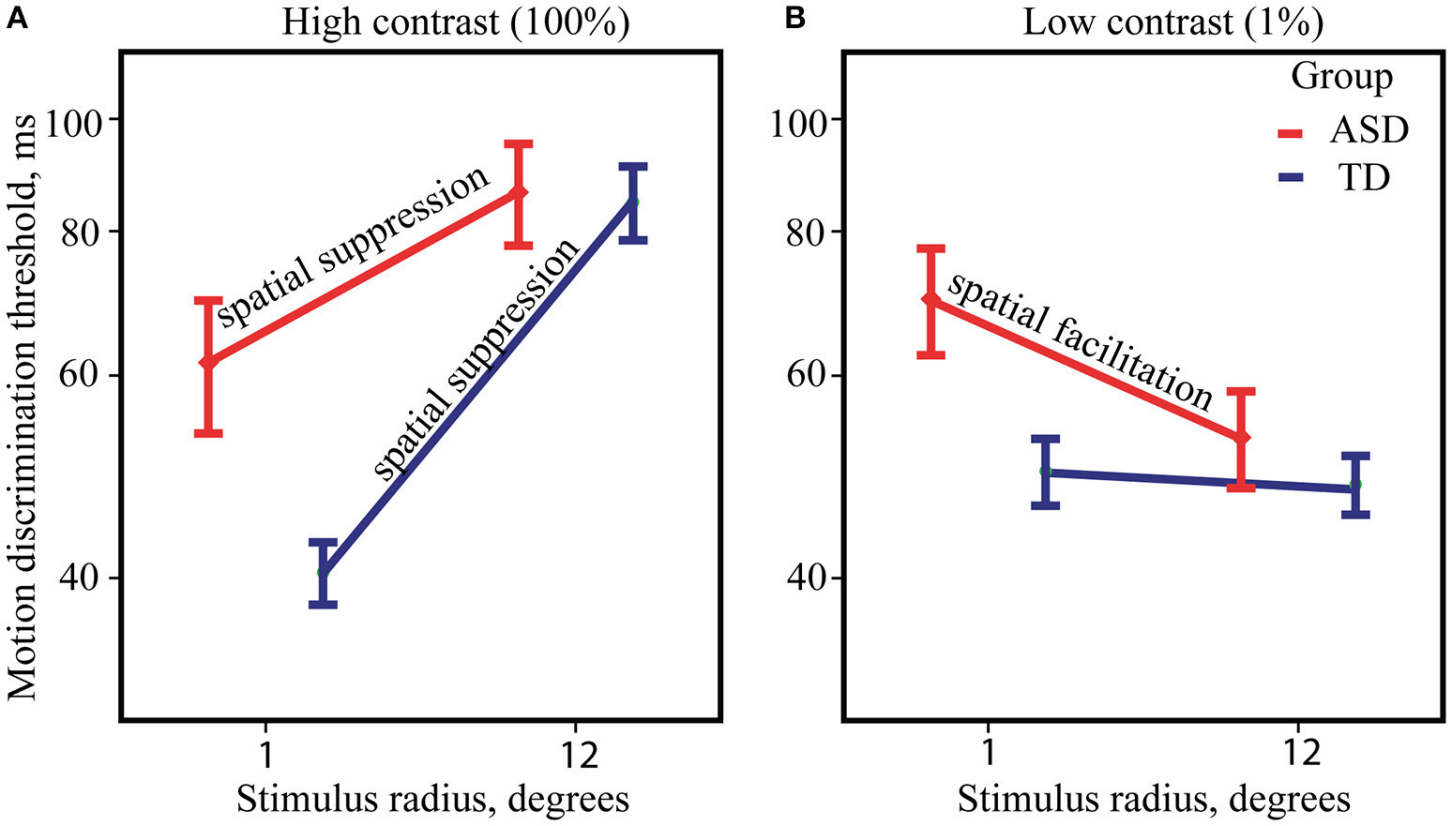
**Size-dependent modulation of motion direction discrimination thresholds**. The mean thresholds for ASD (red) and TD (blue) groups, obtained under high **(A)** and low-contrast conditions **(B)**. Note that for demonstration purpose, threshold values are represented on y-axis in milliseconds along the log-transformed scale.

ANOVA analysis revealed a strong effect of Size [*F*_(1, 82)_ = 157.89, *p* < 0.0001, η^2^ = 0.658] that was due to the higher motion direction discrimination thresholds for large than for small high-contrast drifting gratings. The Group effect was not significant [*F*_(1, 82)_ = 1.17, *p* = 0.28, η^2^ = 0.014] suggesting that on average the ASD individuals have neither superior nor inferior ability to detect direction of motion for briefly presented high-contrast stimuli. There was also a significant Size by Group interaction [*F*_(1, 82)_ = 10.28, *p* = 0.002, η^2^ = 0.111], that is pictured in Figure 2A. Although, the effect of the stimulus size was highly significant in both the ASD [*F*_(1, 39)_ = 40.15, *p* < 0.0001, η^2^ = 0.507] and the TD [*F*_(1, 43)_ = 135.66, *p* < 0.0001, η^2^ = 0.759] groups, it was substantially reduced in participants with ASD as compared with TD participants. The atypically reduced spatial suppression in the ASD was paired with slightly decreased sensitivity to motion direction of small high-contrast stimuli [ASD vs. TD: *F*_(1, 82)_ = 4.84, *p* = 0.03, η^2^ = 0.056], while their thresholds for large stimuli did not differ from normal [ASD vs. TD: *F*_(1, 82)_ = 0.12, *p* = 0.73, η^2^ = 0.001].

#### Low-contrast stimuli

Figure 2B summarizes ANOVA results for low-contrast stimuli. The main effect of Size was small but significant [*F*_(1, 82)_ = 4.57, *p* = 0.035, η^2^ = 0.053] showing the presence of the SF effect, i.e., an improvement in motion perception with increasing size of low-contrast stimuli. The effect of Group [*F*_(1, 82)_ = 2.64, *p* = 0.108, η^2^ = 0.031] was not significant, suggesting that motion discrimination thresholds for low-contrast stimuli on average is similar among ASD and TD individuals. However, significant Group by Size interaction [*F*_(1, 82)_ = 4.04, *p* = 0.048, η^2^ = 0.047] pointed to significantly stronger SF effect in the ASD as compared to the TD group. *Post-hoc* analysis revealed notable improvement of duration thresholds for the large comparing to the small stimuli only in the ASD group [*F*_(1, 39)_ = 10.35, *p* = 0.003, η^2^ = 0.210] and absence of discernible effect in the TD group [*F*_(1, 43)_ = 0.01, *p* = 0.93, η^2^ < 0.001]. Abnormally strong facilitating effect of increasing stimulus size in the ASD group was accompanied by a slightly lower sensitivity to motion direction of small stimuli [ASD vs. TD: *F*_(1, 82)_ = 5.29, *p* = 0.024, η^2^ = 0.061], while there was no significant difference in the thresholds for large stimuli between the groups [ASD vs. TD: *F*_(1, 82)_ = 0.10, *p* = 0.75, η^2^ = 0.001].

#### SS and SF indexes

The SF and the SS indexes are usually calculated as the difference between log-transformed motion direction discrimination thresholds for large and small stimuli under low- and high-contrast condition, respectively. The average indexes of SS and SF are expected to be of opposite signs, with positive values characterizing the SS at high contrast and negative—the SF at low contrast. Note that for both contrast conditions equally, more negative indexes reflect enhanced facilitation and reduced suppression, and, vice versa, more positive values signify a shift of a balance toward relatively greater suppression and lower facilitation. However, individual variability in the SS and the SF strength computed this way may be seriously biased. Indeed, although the SS and the SF indexes are expected to assess the influence of the mechanism operating for the larger stimuli, these indexes in our ASD group correlated only with the motion discrimination thresholds for the small, but not for the large stimuli (see Table 2). This pattern of correlations suggested that in addition to the effect of the increasing stimulus size, the SS and the SF indexes were affected by individual sensitivity to the small moving stimuli. A more appropriate way to extract the unique effect of increased stimulus size on motion discrimination was to compute the residual values of the thresholds for a large stimulus size after partialling out the effect of the threshold for the small stimuli using regression analysis. The SS and SF indexes, calculated this way, correlated only with psychophysical thresholds for the larger stimuli in the both groups (Table 2). Therefore, in all further correlation analyses we used the SS and the SF indexes calculated as the standardized residual values of the thresholds for the large stimuli.

**Table 2:** **Correlations between the psychophysical thresholds and SS and SF indexes**.

**Thresholds**	**ASD**	**TD**	**ASD**	**TD**
	**Methods of indexes calculation**			
	**Subtraction**		**Residuals**	
**High contrast**	**SS index**			
Small	–0.59^**^	–0.39^*^	0.02	0.03
Large	0.07	0.59^**^	0.66^**^	0.88^**^
**Low contrast**	**SF index**			
Small	–0.56^**^	–0.72^**^	0.01	–0.07
Large	0.26	0.46^**^	0.76^**^	0.99^**^

The indexes were calculated either as the subtraction between thresholds for large and small stimuli (Subtraction) or as the residuals for the large stimuli after partialling out its correlation with small stimuli' thresholds with the regression analysis (Residuals). Asterisks denote the significance of correlation coefficient

* *p < 0.01*,

** *p < 0.001*.

### The relations between SS and SF indexes

**Figure 3:**
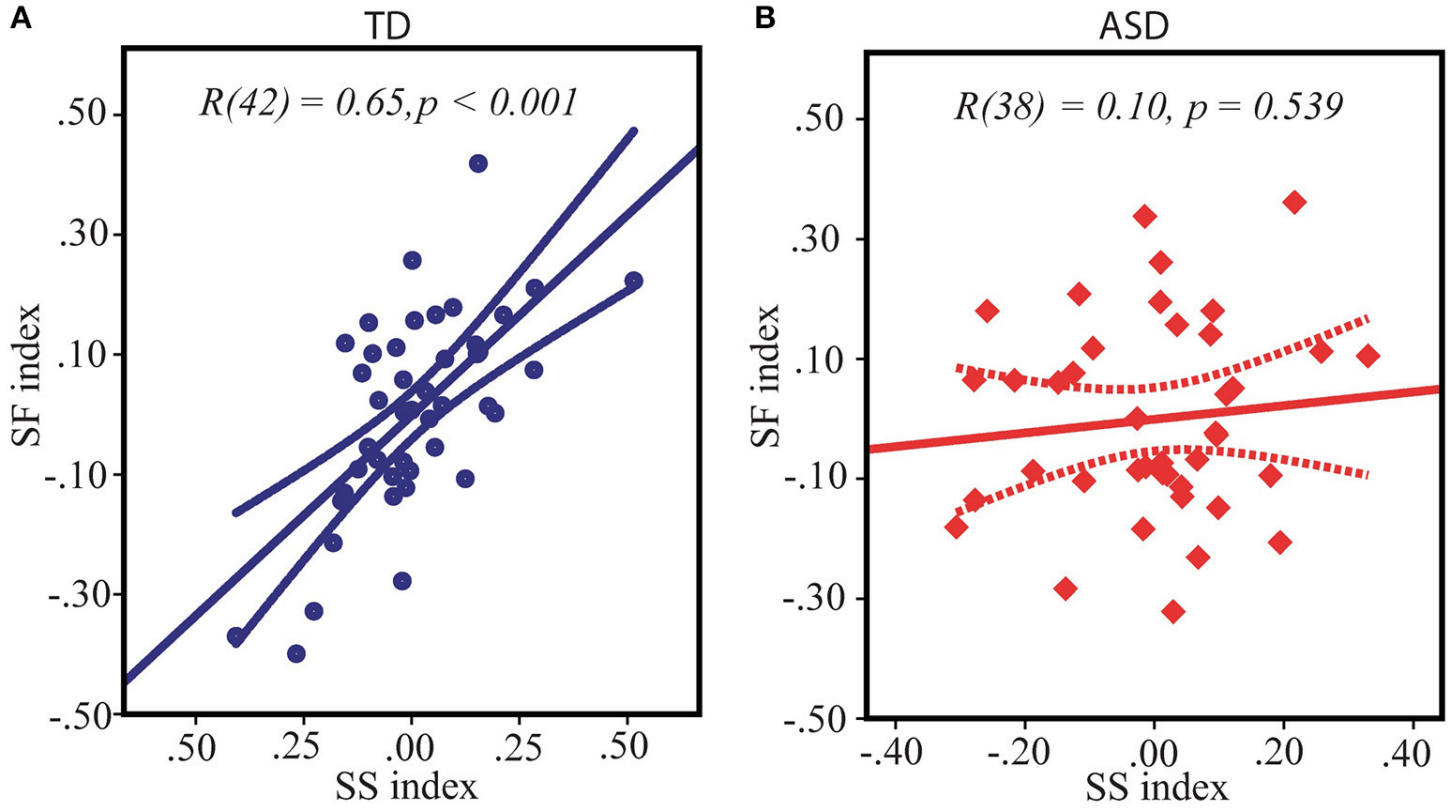
**Relation between spatial suppression and spatial facilitation indexes in TD (A)** and ASD **(B)** children. Note that although the average indexes of contextual modulation assessed under high and low-contrast conditions respectively are expected to be of opposite signs, for both conditions equally the greater values signified a shift of a balance toward relatively stronger suppression and weaker facilitation, and, vice versa, the lower individual indices reflected enhanced facilitation and reduced suppression. Therefore, positive correlation between spatial suppression and spatial facilitation found in TD children indicates that on the individual level stronger spatial suppression under high contrast is associated with weaker facilitation under low contrast condition.

As reviewed in the introduction, both the SS and the SF indexes are influenced by the E/I balance in primary visual areas, suggesting the interrelation between SS and SF. Indeed, in our TD participants the SS and the SF indexes obtained under two different contrast conditions were strongly directly interrelated: *r*_(42)_ = 0.65, *p* < 0.0001 (Figure 3A). Noteworthy, this correlation was missing in the ASD group: SS vs. SF: *r*_(38)_ = 0.10, *p* = 0.54 (Figure 3B), with correlation coefficient being significantly different from that in the TD group: Z = 2.98, *p* = 0.003. This fact might indicate that higher inhibition from the “near surround” typically affected subject's perception of large moving stimuli even under low-contrast condition, downshifting a strength of SF effect in TD individuals but not in ASD participants.

### SS/SF indexes and the severity of autism

**Figure 4:**
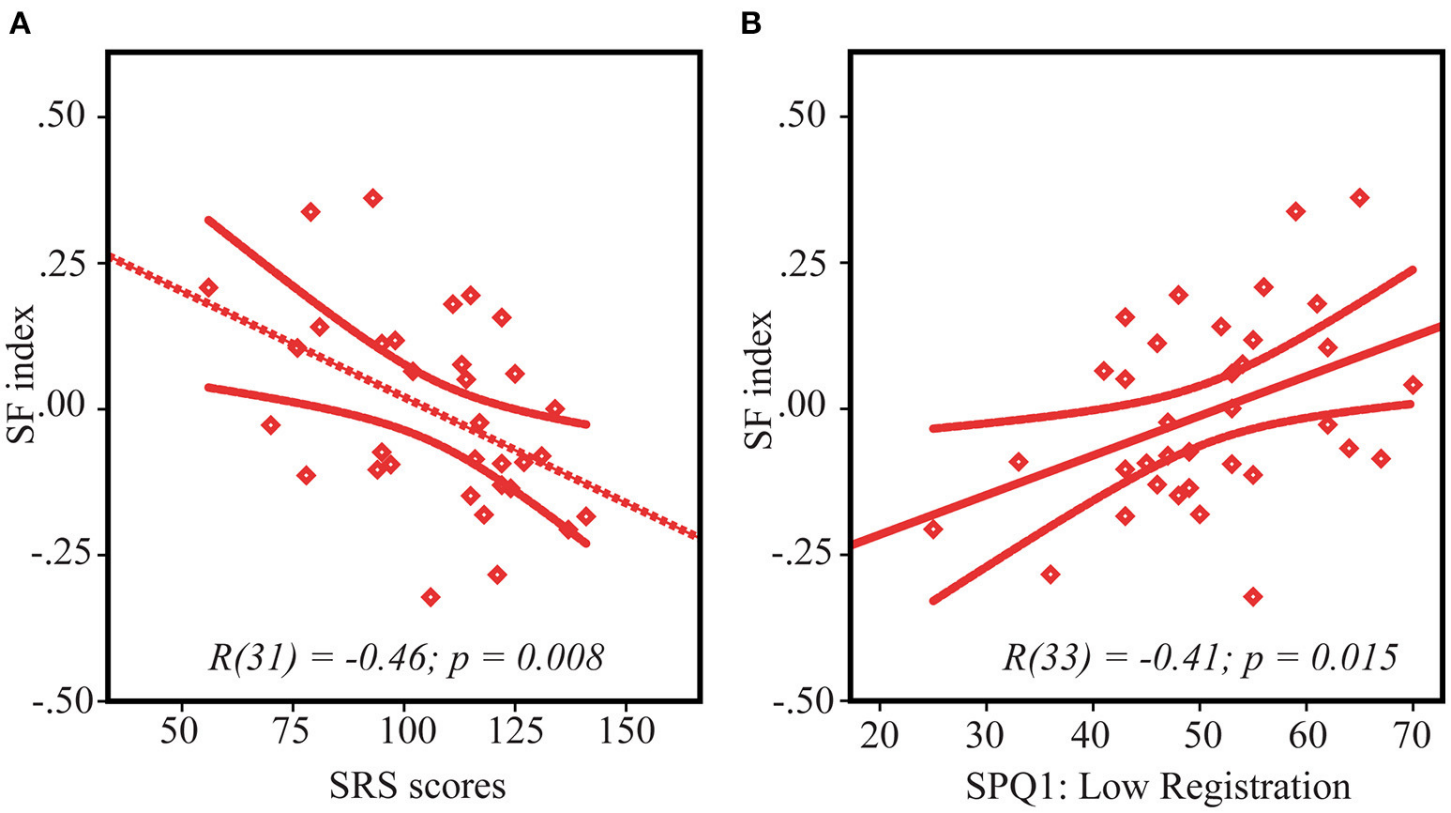
**Spatial facilitation and ASD symptoms**. Correlation between SF index and SRS scores **(A)** and sensory processing abnormalities—low registration scores **(B)** in ASD children. Note that lower SF scores indicate stronger spatial facilitation. Since higher scores on SRS and lower scores on low registration correspond to higher symptoms severity, the direction of their relationships with SF is the same, despite an opposite sign of their correlations.

SS index correlated with the SRS scores in neither the ASD nor the TD groups (both /*r*/s < 0.21, both *p*'s > 0.24). However, the SF index significantly correlated with the SRS scores in the ASD group [*r*_(31)_ = −0.46, *p* = 0.008] in such a way that greater facilitative effect of increasing size was associated with greater autism severity (Figure 4A). Noteworthy, this association is unlikely to be explained by generally impoverished performance in more severely affected children, since the motion discrimination thresholds did not correlate with the SRS scores [small stimuli: *r*_(31)_ = 0.09, *p* = 0.60, large stimuli: *r*_(31)_ = −0.30, *p* = 0.09, negative sign of the correlation indicates that the participants with better performance had a tendency to have higher SRS scores, but not vise versa]. In the TD group the correlation between SF index and SRS was absent [*r*_(37)_ = 0.15, *p* = 0.39].

### SS/SF indexes and sensory processing abnormalities

ASD pariticipants had higher rates of sensory processing dysfunction than the children without autism on all 4 categories/quadrants of the SPQ (all *t*'s > 4.5, all *p*'s < 0.0001, Table 1). In the TD group both the SS and the SF indexes did not significantly correlate with any of the 4 quadrants measures [*r*_(40)_ < 0.25, *p* > 0.15]. The SF index in the ASD group significantly correlated only with Low Registration quadrant [*r*_(33)_ = 0.41, *p* = 0.015 (Figure 4B), all other *r*'s < 0.25]. According to Dunn's Model of Sensory Processing in daily life (Dunn, 1997, 1999) this behavioral index reflects a combination of high sensory thresholds and passive response to everyday events. Low registration scores were worse in those children and adolescents with ASD who demonstrated a higher SF effect, i.e., relatively enhanced motion perception of large low-contrast stimuli, comparatively to small ones. To ensure that the observed correlation did not originate from generally poor sensitivity to low-contrast stimuli in more compromised participants with ASD, we evaluated correlation between the low registration scores (SPQ1) and motion duration thresholds under a low-contrast condition. Neither of these correlations reach significance level [low-contrast small vs. SPQ1: *r*_(33)_ = −0.16, *p* = 0.35, low-contrast large vs. SPQ1: *r*_(33)_ = 0.21, *p* = 0.23].

## Discussion

Our main findings on the SS and the SF effects in children and adolescents with ASD can be summarized as follows. *Firstly*, for the high-contrast stimuli we found slightly worse sensitivity to moving stimuli of a small size in participants with ASD, while the suppressive effect of increasing stimulus size was reduced in the ASD compared to the TD group. *Secondly*, for the low-contrast stimuli, the elevated motion discrimination thresholds to small stimuli in participants with ASD were paired with atypically strong spatial facilitation, i.e., size-dependent improvement in discrimination of motion direction. *Thirdly*, the SS and the SF indexes were strongly correlated in the TD group, while this correlation was absent in the ASD group. *Fourthly*, in the participants with ASD the SF, but not the SS, correlated with the severity of autism and with sensory processing difficulties occurring in daily life. Noteworthy, the motion discrimination thresholds did not correlate with these measures, arguing against the possibility that these findings are driven by poor performance in more severely affected participants with ASD.

This pattern of results suggests that the center-surround interaction during processing of moving stimuli is altered in individuals with ASD in the direction of atypically strong facilitative and weak inhibitory influences from the surround of the “non-classical” receptive field.

### Comparison with results of Foss-Feig et al. (2013)

Since our findings on motion direction discrimination thresholds and the effect of stimulus size/contrast on perception of motion direction in ASD are partially inconsistent with the previous report of Foss-Feig et al. (2013), we compare in more details the results of the two studies below.

Children with ASD in Foss-Feig et al. (2013) study exhibited an atypical superiority of motion direction perception, which was evident only for high-contrast but not low-contrast stimuli. They also showed an enhanced spatial facilitation for low-contrast stimuli of large size with unaltered spatial suppression effect in a high-contrast condition. Our data demonstrated an abnormally strong spatial facilitation in participants with ASD, but do not contain any evidence in favor of “substantial and unexpected enhancement” in perceiving direction of motion for high-contrast stimuli in this population. In addition, unlike Foss-Feig et al. we did find an abnormally weak spatial suppression in individuals with ASD, whose motion direction discrimination thresholds were less affected by the increase in size of the high-contrast stimuli. This deficit in spatial suppression is theoretically expected since it characterizes elderly individuals and those with schizophrenia and depression (Betts et al., 2005; Tadin and Blake, 2005; Tadin et al., 2006; Golomb et al., 2009), who, similarly to ASD participants, are likely to have a deficit in cortical inhibition.

Given that the experimental paradigm used in our study was similar to that used by Foss-Feig et al. (2013), the differences in the findings of the two studies can be attributed to the differences in characteristics of experimental samples. Foss-Feig et al. examined extremely high-functioning children and adolescents with ASD (mean IQ = 116, which tended to be significantly higher than the respective mean score of 106 in the TD group, *p* = 0.09). Distribution of IQ scores in our ASD sample (mean IQ = 89; range 63–123) was more representative of that in a more general ASD population, which is characterized by the frequent presence of cognitive disabilities (Charman et al., 2011). The finding of unchanged or even slightly increased motion direction discrimination thresholds for the high-contrast stimuli in children and adolescents with ASD in our study could potentially be explained by a strong effect of intellectual disability and related difficulties with sustaining attention and motivation. These high-order cognitive factors could blur the perceptual advantage in the motion direction discrimination found by Foss-Feig et al. in their ASD sample with above average IQ. However, the results of the current study did not show a correlation between IQ and psychophysical thresholds for the detection of motion direction of either high-contrast or low-contrast stimuli of any sizes, as well as SS and SF indexes in the ASD group (*r*'s < 0.2, *p*'s > 0.2) suggesting that this was not the case.

An alternative explanation of inconsistency in findings between the two studies refers to the genuine effect of different sub-types of autism developmental disorder. Specifically, the presence of atypically high or atypically low sensitivity to motion in people with ASD might depend on severity/quality of the disorder, e.g., on the presence or absence of developmental language delay, which was shown to be strongly associated with development of non-verbal intellectual abilities (Wodka et al., 2013). Indeed, the relationship between language abilities and motion perception has been repeatedly demonstrated (Gepner and Mestre, 2002; Takarae et al., 2004, 2008; Samson et al., 2012). In particular, Takarae et al. reported that ASD groups with and without early speech delay perform differently in visual saccade and motion discrimination tasks (Takarae et al., 2004, 2008). In addition, motion processing deficits were relatively specific to the group with early language delay. In our ASD sample comprising participants with different IQ levels, children with speech delay were relatively common (71% according to the available medical records). On the other hand, one might expect that the majority of the high functioning participants with ASD in Foss-Feig et al. study had no speech delay, although the exact proportion is unknown. These between-sample differences in the developmental history of autistic disorder can be linked to different patterns of brain maturation in less and more severely affected individuals which could also explain why a weakening of classic spatial suppression effect found in the current study was absent in Foss-Feig et al. sample.

### SS and SF effects in the TD children

The current study's findings in TD participants are fully consistent with previous literature. Firstly, TD participants exhibited highly reliable spatial suppression at high contrast. The impoverished motion perception of bigger stimuli at higher contrasts is in line with numerious evidence from the human and animal studies (Pack, 2004; Tadin and Lappin, 2005; Tadin et al., 2011). Secondly, there were no psychophysical manifestations of either spatial suppression or spatial facilitation in the TD group under low-contrast condition. This results can be related with previous literature, taking into account that spatial summation effects are strongly dependent on the exact size and contrast of moving stimuli (Tadin and Lappin, 2005; Tadin et al., 2006; Foss-Feig et al., 2013). The only study (Foss-Feig et al., 2013) which examined SF in TD children and adolescents showed that although a psychophysical spatial summation effect was quite strong with increasing stimulus size from small (1° radius) to medium (2.5° radius), the effect disappeared for the larger stimuli (6°). Since our study utilized small stimuli with a radius of 1° and large stimuli with a radius of 12°, which was even bigger than that used by Foss-Feig et al. our results show high correspondence with those of the previous study.

Foss-Feig et al. (2013) suggested that the disappearance of psychophysical signs of spatial facilitation with increasing stimuli size at low contrast seen in TD children might be caused by the influence of neurophysiological process underlying spatial suppression and operating for large stimuli even at low contrast. This suggestion is well in line with our findings. In our study, the SS and SF indexes were highly correlated in our TD group. Specifically, we have found that TD participants with the largest SS effect of the increasing stimulus size at high contrast demonstrated also the weakest spatial facilitation with increasing stimulus size at low contrast. This interdependence was absent in ASD participants.

The strong correlation between the SS and the SF phenomena, observed in the TD group, might be explained using a model based on the data obtained from primate neurophysiology (Angelucci and Bressloff, 2006; Schwabe, 2006). According to this model, both SS and SF effects arise from the excitatory feedback projections from “motion-sensitive” area MT to the representation of the visual field periphery in primary visual cortex (V1). The purely excitatory horizontal connections from the “far surround” neurons target both excitatory and inhibitory neurons in the “near surround” of the receptive field (RF) center. The model of Angelucci et al. together with numerous experimental evidences (Kapadia et al., 1999; Mizobe et al., 2001; Hess et al., 2003), suggest that “suppressive far surround” is not always suppressive, but can also facilitate the response of the RF center, depending on the amount of excitatory drive to the local inhibitors. Given that an excitatory threshold for local inhibitory neurons is higher than that for excitatory one, the excitatory feedback from the MT engages the V1 local inhibitory circuitry much more under condition of high comparing to low contrast moving grating of a large size. An increased inhibition from the “near surround” is the main cause of paradoxically increased motion duration thresholds for high-contrast stimuli of a bigger size. On the contrary, when stimulus contrast is low, the excitatory “near surround” driven by the weaker excitatory feedback signal prevails over the local inhibitory one causing the facilitation of movement detection for large low-contrast stimuli as compared to small ones. Thus, although large moving stimuli of either contrast activate both excitatory and inhibitory “near surround” input to classic RF in V1, the balance between inhibitory and excitatory inputs is shifted in opposite directions for high and low contrast large stimuli. This scenario predicts that at the behavioral level the effectiveness of size-dependent modulation for low and high contrast stimuli (the SS and the SF indexes) must be linked to one another, a prediction which is confirmed by our finding in the TD group (Figure 3A).

The highly significant relationships between magnitude of the SF and the SS effects in the TD children, may originate from individual variability in the strength of feedback connections from MT to V1. Indeed, a heightened feedback signal increases, although to a different degree, the contribution of inhibitory input from the “near surround” for both high-contrast and low-contrast conditions. This results in strengthening a psychophysical effect of spatial suppression and attenuation of spatial facilitation. An alternative explanation of the observed correlation between SS and SF effects in our TD participants is the inter-individual variability in the effectiveness of the local inhibitory circuitry of the primary visual cortex. An individually heightened excitability of inhibitory circuitry may result in the higher ratio of suppressive/facilitative influences from the “near surround” both at high and at low contrast; thus increasing spatial suppression and decreasing spatial facilitation indexes at the behavioral level.

### Increased SF and decreased SS in ASD

The lack of correlation between the SS and SF indexes in children with ASD (Figure 3B) paired with their abnormally strong SF and weak SS effects (Figure 2) suggests that in a substantial proportion of children with ASD the surround inhibition at low contrast is drastically reduced giving way to purely facilitative influences from a periphery of the visual field.

There may be at least two hypothetical mechanisms underlying the imbalance between facilitative and suppressive surround influences during motion perception in ASD. One possibility, suggested by single cells recordings, is that a reduced surround inhibition in our ASD group is caused by the atypically weak feedback signal from MT. However, arguing against this suggestion, fMRI studies with ASD individuals found that their activation of MT/V5 motion area was not altered during forced-choice motion discrimination task (Robertson et al., 2014), or was even atypically enhanced during passive viewing of drifting ripple pattern (Schwarzkopf et al., 2014; Takarae et al., 2014). A more feasible explanation based on animal studies suggests the low effectiveness of the “near-surround” inhibitory signal in primary visual cortex of individuals with ASD. The weak local inhibitory signal is still sufficient to cause surround inhibition in case of highly salient moving stimulus of large size and high contrast but is not enough to suppress the neurons within the classical RF when a stimulus contrast is low.

An assumption that local inhibitory signaling may be affected in the autistic brain is broadly consistent with the prior evidence from the postmortem studies of human brain tissue (Hashemi et al., 2016) as well as with the data obtained in animal models of ASD (for review see, Pizzarelli and Cherubini, 2011). These studies jointly suggest that deficit in GABAergic transmission across multiple brain areas may contribute to diverse behavioral symptoms of ASD. However, psychophysical and neuroimaging studies of ASD has paid surprisingly little attention to the low-level visual functions that are known to depend on the inhibitory “near surround” circuitry of V1. Notably, the rare available evidence is in accord with putatively deficient local inhibition in primary visual cortex in a proportion of ASD individuals (Robertson et al., 2013, 2016; Stroganova et al., 2015; Sysoeva et al., 2016).

To sum up, the E/I imbalance in V1 circuitry caused by reduced inhibitory input from the “near surround' might contribute to the observed SS and SF abnormalities in our participants with ASD. The difference in the results of the present study and that of Foss-Feig et al. (2013) could be explained by the less pronounced E/I balance abnormalities in the exceptionally high-functioning ASD population investigated by these researchers. In the latter population, a relatively less compromised inhibition from the near surround could have only a latent effect on psychophysical surround suppression at high contrast when excitatory drive to local inhibitory circuitry in V1 is strong. However, at low contrast and weak excitatory drive, the inhibitory deficit might become apparent as an abnormally enhanced psychophysically measured spatial facilitation. Thus, seemingly different results from high-functioning ASD participants (Foss-Feig et al., 2013) and those from a general ASD population (current study) may reflect a lesser severity of the inhibitory deficit in V1 in the former case.

### SF and the severity of autism

In our ASD sample strength of SF was correlated with the prevalence of autistic symptomatology (Figure 4A) and sensory processing abnormalities described in sensory profile questionnaire as “Poor registration abilities” (SPQ1: Low Registration, Figure 4B). Since the autism severity measured by SRS as well as severity of the sensory difficulties (“poor registration”) correlated with the SF index, but not with the motion direction discrimination thresholds themselves, this correlation is unlikely to be driven by poor performance of the more severely affected children. Notably, previous studies also linked alternations of elementary visual functions to severity of autism (Robertson et al., 2014; Stroganova et al., 2015, but see Foss-Feig et al., 2013) and explained this link through the presence of a more pronounced and widespread inhibitory deficit in the more severely affected individuals with ASD. However, it is difficult to envision how such a mechanism alone could account for our finding of this specific relation of autism symptomatology to atypically enhanced spatial facilitation, but not to reduced spatial suppression in our ASD participants.

According to Dunn (1997, p. 31) the “poor registration ability” implies that “children …can be withdrawn and difficult to engage or self-absorbed. Those who are withdrawn are easily exhausted, appear apathetic, and need highly salient stimuli to engage them.” The abnormally increased spatial facilitation may represent a way in which the developing ASD brain compensates for a withdrawal from sensory environment, through emphasizing some features of otherwise poorly discernible low contrast moving stimuli. Neural facilitation is known to preferentially occur with near-threshold visual stimuli, when the visual system attempts to integrate insufficient information across visual space and in doing so relies upon inter-cortical interactions and increased feedback from the near- and/or far-extrastriate regions (Nauhaus et al., 2009). These compensatory mechanisms may have neurobiological basis that is different from the inhibitory deficit. Absence of the correlation between the reduced SS and the enhanced SF in our participants with ASD also indicate that facilitatory influences in the visual cortex can be increased independently from the deficit in surround inhibition. It is worth noting that, in addition to inhibitory deficit, the increased facilitation may contribute to the reduced divisive normalization (Rosenberg et al., 2015) and result in the unreliable and noisy electrophysiological responses (Dinstein et al., 2012) observed in autism.

## Conclusions

This study was motivated by the hypothesis of the reduced inhibitory signaling as the main neurobiological cause of abnormalities of the elementary visual perceptual functions in ASD. We analyzed well-studied psychophysical phenomena of spatial suppression and spatial facilitation that critically depend on excitation and inhibition balance in visual cortex. Our study confirmed the previous results (Foss-Feig et al., 2013) on the presence of abnormally enhanced spatial facilitation in children and adolescents with ASD. Congruent findings from the different ASD samples are especially important given a generally poor replication of psychophysical results (Kaiser and Shiffrar, 2009; Simmons et al., 2009) which originates from enormous heterogeneity of pathophysiological causes of ASD. From this perspective, the reliable enhancement of spatial facilitation in individuals with ASD that is not related to degree of their cognitive disturbances suggests that an increased spatial summation of a weak visual signal is a common feature of ASD clinical phenotype. Strong direct relationships that we found between the spatial facilitation and the severity of autism symptomatology strengthen this conclusion.

Beyond a mere replication of the prior finding, our results bear evidence of an abnormally weak spatial suppression in ASD—the result that was expected given multiple indications of reduced functionality of inhibitory signaling in ASD (Pizzarelli and Cherubini, 2011). The absence of the typical link between SS and SF indexes in our participants with ASD also implies that in their visual cortex the neural inhibition is reduced and is not sufficiently strong to be recruited during stimulation with weak low-contrast stimuli. Our results thus substantiate the proposal of Foss-Feig et al. (2013) regarding hypofunctional inhibitory transmission in visual areas as one of the possible sources of the enhanced SF in ASD. Nonetheless, unexpectedly we found that the SF rather than the SS was linked to autism symptoms, emphasizing a role of heightened excitatory influences by themselves in observed abnormalities in low-level visual phenomena found in ASD.

To sum up, our results add to a growing literature on atypical low-level visual functioning in children and adolescents with ASD that is likely to be driven by an altered E/I balance in the visual cortex.

## Author contributions

TS, EO, and OS substantially contributed to the conception and design of the work, as well as to analysis and interpruption of the data; IG, MD, and OS substantially contributed to the data acquisition and analysis. All authors participated in drafting the work or revising it critically for important intellectual content, approved the final version for publication, and provided an agreement to be accountable for all aspects of the work in ensuring that questions related to the accuracy or integrity of any part of the work are appropriately investigated and resolved.

## Funding

The study has been supported by Russian Science Foundation grant #14-35-00060 and the charity foundation for autism “Way out.”

### Conflict of interest statement

The authors declare that the research was conducted in the absence of any commercial or financial relationships that could be construed as a potential conflict of interest.
